# An Efficient Apparatus for Rapid Deoxygenation of Erythrocyte Concentrates for Alternative Banking Strategies

**DOI:** 10.1155/2013/896537

**Published:** 2013-03-10

**Authors:** Lello Zolla, Angelo D'Alessandro

**Affiliations:** Department of Ecological and Biological Sciences, Tuscia University, Largo dell'Università, snc, 01100 Viterbo, Italy

## Abstract

Erythrocyte concentrates (ECs) stored for transfusion purposes still represent a lifesaving solution in a wide series of clinically occurring circumstances, especially for traumatized and perioperative patients. However, concerns still arise and persist as to whether current criteria for collection and storage of ECs might actually represent the best case scenario or there might rather be still room for improvement. In particular, the prolonged storage of EC has been associated with the accumulation of a wide series of storage lesions, either reversible (metabolism) or irreversible (protein and morphology). Independent laboratories have contributed to propose alternative strategies, among which is the introduction of oxygen removal treatments to ECs. Convincing biochemical and preliminary clinical evidences have been produced about the benefits derived from the introduction of this practice. We, hereby, propose a rapid, efficient, and time-effective strategy for blood deoxygenation which might fit in current EC production chain. The proposed strategy resulted in the complete deoxygenation of red blood cell hemoglobin (pO_2_ < 0.0021 mmHg). A preliminary small-scale study about the application of the present method resulted in reduced hemolysis, decreased vesiculation, and limited alterations to the red blood cell morphology, as gleaned from flow cytometry and scanning electron microscopic analyses. Further in-depth and larger-scale investigations are encouraged.

## 1. Introduction

Erythrocyte concentrates (ECs) are still the most widely transfused blood-derived therapeutic worldwide, as many million units of blood are collected, and million units of RBCs are administered to millions of patients every year [1, 2]. Currently, accepted European Council guidelines indicate that ECs may be stored for up to 42 days under controlled conditions before transfusion [3]. Nevertheless, recent retrospective and controversial studies have brought about concerns on the suitability of longer stored EC units for transfusion purposes [4, 5]. It has indeed been stressed that the risk of exposure to long-stored red blood cells (RBCs) is exacerbated when dealing with certain categories of recipients, such as traumatized, postoperative, and critically ill patients [5]. However, it should be worth mentioning that early results from randomized double-blind clinical prospective trials have not hitherto indicated any statistically significant disadvantage of the administration of longer stored units in comparison to fresher blood [6, 7]. 

While the likelihood of untoward effects related to the transfusion of older RBC units is still a matter of debate and of clinical investigations, what is now known for certain is that storage affects biochemical and biological properties of RBCs, and the extent of these accumulating changes, collectively known as “storage lesions,” is proportional to the duration of the storage period [8–12]. Storage lesions include alterations to either morphology (shape changes leading from a discoid to a spherocytic phenotype) or functionality (metabolism and oxygen delivery capacity through an increase in oxygen affinity mediated by a rapid fall in 2,3-diphosphoglycerate concentrations [8–13]). Further lesions occur in stored RBCs which are reversible to some extent, such as potassium leakage to the supernatant and depletion of ATP and DPG stores, while others are not, such as the alteration of lipids and membrane proteins (membrane protein fragmentation and migration to the membrane and/or vesiculation of subsets of structural or antioxidant proteins [9]), which results in more rigid cell structures, increased osmotic fragility, higher haemolytic rates, phosphatidylserine exposure to the outer membrane leaflet, increased vesiculation rate, and reduced oxygen off-loading capacity [8–12, 14, 15]. 

Membrane protein fragmentation [9, 13], storage time-dependent migration of cytosolic proteins to the membrane [9, 14, 15], and increased oxidative stress-related parameters [9, 16] have been also reported to correlate with storage duration. Biochemical studies explicitly suggested that there is considerable room for improvement in the field of RBC biopreservation, especially when considering that the lifespan of RBCs *in vivo* is approximately 120 days [17]. One major phenomenon seems to lie at the root of storage lesions to RBCs: oxidative stress [9, 13, 16]. In order to cope with oxidative stress triggering phenomena, alternative RBC storage strategies have been recently proposed, such as the addition of higher loads of antioxidants (vitamin E, C, and beta-carotene) in additive solutions for storage purposes [18] or anaerobic storage [13, 19–21]. While the former strategy has been designed as to counteract oxidative stress arising over prolonged storage, the latter has been thought as to prevent overproduction of reactive oxygen species (ROS) through the elimination of the main substrate, oxygen.

The anaerobic approach (pO_2_ < 4% - patent WO/1996/039026) has been reported to deliver ECs with hemolysis below 0.8% and *in vivo *survival at 24 h upon transfusion above the 75% threshold [19–21]. Independent studies from our group have evidenced that storage under helium also reduced the extent of membrane protein fragmentation or aggregation phenomena of nonleukofiltered erythrocyte concentrates [13]. However, it should be appreciated that these optimistic results are currently undergoing further clinical testing through prospective trials. The clinical milieu has so far looked at the “anaerobic perspective” with diffidence, mainly because the recently proposed protocols implied the introduction of new costly and time-consuming steps in the EC production chain. In other terms, the alternative “anaerobic perspective” appeared not to be optimized for cost/benefits or cost/effectiveness considerations.

In this view, we hereby propose a rapid strategy for cost- and time-effective deoxygenation of ECs and provide details about the likely frame of steps in which this method might be safely and efficiently introduced in clinical routine practice in the future.

## 2. Technical Design

### 2.1. Blood Collection

Whole blood (450 mL + 10%) was collected from healthy volunteer donors into citrate phosphate dextrose (CPD) anticoagulant (63 mL; pH 5.6) and leukodepleted. After the separation of plasma by centrifugation, RBCs were suspended in 100 mL of SAG-M (Saline, Adenine, Glucose, Mannitol) additive solution. We studied RBC units collected from 8 donors (male: 4, female: 4, age 45 +11.5 (mean + S.D.)). The present study was approved by the Italian National Blood Centre (Rome, Italy).

### 2.2. Helium Cylinders

High purity helium gas cylinders (99.999% of gas purity, 10 m^3^ each) were obtained from Sol S.p.A. (Pomezia, Italy).

### 2.3. Deoxygenation of Red Blood Cells

ECs were stored into CPD-SAGM-containing plastic bags (Fenwal Italy, Milano, Italy).

**Figure 1:**
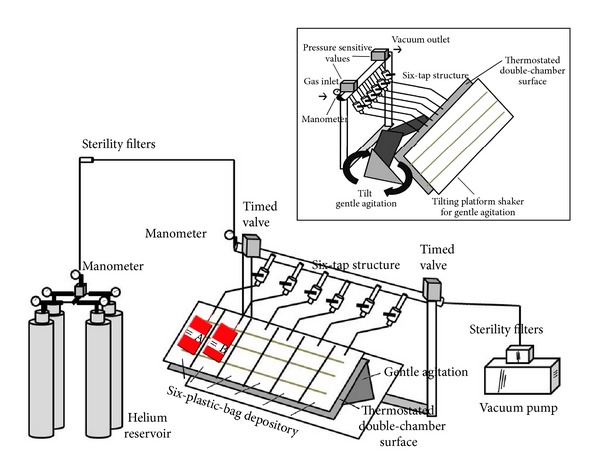
A schematization of the blood deoxygenation apparatus. Gas cylinders provide highest purity helium whose pressure is controlled through a manometer. Gas inlet is regulated in a closed system through a time valve which temporizes inlet and outlet towards a vacuum pump after 5 minutes of gas exchange within the plastic bag. Plastic bags are blocked almost horizontally as to favour gas exchange, through gentle agitation and temperature modulation. Sterility filters ensure the sterility of the whole system, either in the gas inlet or outlet tubing.

An illustration of the deoxygenation apparatus is reported in Figure 1.

Helium from gas cylinders was regulated for 1 bar output pressure through a common manometer and fluxed into the EC units through sterile connection. 

A time valve (standard execution, up to 10 bar; regulation from 1 to 10 seconds) was used to regulate both influx and efflux of helium, through the opening of gas influx for 5 seconds, stabilizing the system for six minutes (in order to allow gas exchange between RBCs in the unit and the gas phase) and the opening of the second valve linked to a vacuum pump aspirating the gas from the unit.

Sterility was further guaranteed by the presence of sterility filters (AcroPak 300-Pall Life Sciences, NY, USA) in between the gas outlet from the cylinder manometer and the inlet tube into the unit and from the unit to the vacuum pump.

Each unit was conditioned with helium through 5 cycles of influx and aspiration via vacuum pump. Each cycle consisted in helium influx, six minutes gas exchange between RBCs in the unit and the gas phase and gas efflux through the opening of the vacuum pump valve.

In order to ease gas exchange, RBC units are placed on a tilting stainless steel plate for gentle agitation, as to prevent hemolysis. The stainless steel surface was thermostated at 37°C, in order to ease oxygen dissociation from hemoglobin [22–24]. Temperature stability was guaranteed by an internal resistance which was used to warm water circulating within a tunnel in the double chamber of the stainless steel plate. 

The apparatus was optimized to perform deoxygenation on six units simultaneously, by means of a six-tap structure (Steroglass; Perugia, Italy) and sterility filters at the end of each tap. Depositories for six independent units were used to block the bags on the stainless steel surface for the duration of the deoxygenation process (30 minutes).

RBC units were stored under standard blood bank conditions (1–6°C) in a closed chamber conditioned with helium for up to 42 days.

### 2.4. Assessment of Conservation of the Deoxygenated Condition

Prior to storage, hemoglobin oxygenation levels were assayed spectrophotometrically through a double-beam spectrophotometer Cary 4 Varian and further tested with dissolved oxygen sensors by tryptophan fluorescence quenching (<0.0021 mmHg) (Steroglass, Perugia, Italy).

**Figure 2:**
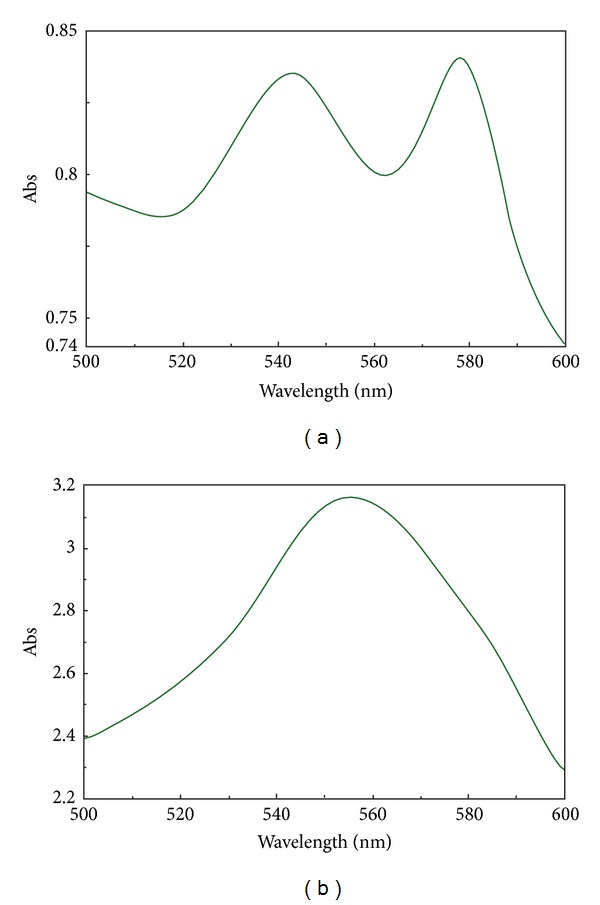
Spectrophotometric absorption of hemoglobin in the 500–600 nm range, prior to (a) or after (b) deoxygenation (30 min, 6 cycles of 5 minutes each). The assays were performed directly on red blood cells within the plastic bag, as described in the paper. No significant scattering is observed.

Since our goal was to test deoxygenation levels, albeit not hemoglobin concentration, we did not need to establish a precise path length, in agreement with the Lambert-Beer law. Conversely, we were interested in performing the assay directly on the blood unit. The internal architecture of the Cary Varian spectrophotometer allowed us to perform the analysis directly on vertically placed units, where blood was allowed to drip on the lateral surface of the plastic bag and the majority of the unit (labels included) was put below the optic path of the laser beam, in order not to disturb the reading. Any effect of the absorbance and scattering of the plastics was excluded in the range of 500 < *λ* < 600 nm. When only a film of blood was visible in the lateral wall of the unit (thus excluding any scattering associated with higher volumes of packed RBCs) it was possible to measure hemoglobin absorbance without any significant scattering within a time window of 1 min.  Figure 2 shows hemoglobin absorbance spectra prior to (a) and after (b) deoxygenation. The (few) inconveniences of such a home-made strategy could be easily overcome by a specifically designed plastic bag with a room of a fixed volume (0.01 cm wide, e.g.). Further testing with dissolved oxygen sensors by tryptophan fluorescence quenching indicated pO_2_ below 1 ppb (<0.0021 mmHg) (Steroglass, Perugia, Italy), below the limit of detection of the instrument.

No bacterial contamination was observed at the end of the storage period in either control or deoxygenated units, as gleaned through MALDI Biotyper analyses [25].

### 2.5. Hemolysis

Hemolysis was calculated following the method by Harboe [26]. Samples were diluted in distilled water and incubated at room temperature for 30 min to lyse red blood cells. Samples from lysed RBCs were diluted 1/300 while supernatants were diluted 1/10 in distilled water. After stabilizing during 30 min and vortex mixing (Titramax 100, Heidolph Elektro, Kelheim, Germany), the absorbance of the hemoglobin was measured at 380, 415, and 450 nm (PowerWave 200 Spectrophotometer, Bio-Tek Instruments, Winooski, Vermont, USA). The mean blank was subtracted, and the corrected OD (OD*) was calculated as follows: 2 × OD_415_ − OD_380_ − OD_450_.

### 2.6. Flow Cytometry

RBC supernatants of control and deoxygenated units were collected upon 42 days of storage for flow cytometry-based analyses of RBC shed microvesicles. RBC microparticles released during storage were separated from RBCs by centrifugation of RBCs transferred into 50 mL tubes for 10 minutes at 1000 ×g at room temperature. The supernatant was recentrifuged for 5 minutes at 2000 ×g at room temperature. The resulting supernatant containing RBC microparticles was collected and centrifuged for 30 minutes at 18,000 ×g at 4°C. The centrifugation speed of 18,000 ×g was selected on the basis of the method optimized by Rubin et al. [27].

 The morphology of the cells and microparticles was assessed by a FACScalibur (Becton-Dickinson, USA). A standard method for approximate quantitation of RBC microparticles on the basis of their relative size and shape was applied [27, 28]. Although the method holds some pitfalls, which could be partly overcome through the use of specific antibodies against microvesicle markers (e.g., CD132 and CD235a) or annexin V (against phosphatidylserine), the presently exploited method still allows obtaining an indicative idea about the relative quantities of microparticles in a given RBC concentrate supernatant. Analyses were conducted using the instrument software by counting events in a 5-minutes time window within the gated area [27, 28]. Events were analysed on the basis of side scatter and forward scatter, as compared against a flow cytometry size calibration kit (Invitrogen, Eugene, OR) containing beads of different diameter, from ~1 *μ*m to ~1.4 *μ*m (RBC microparticles are smaller than those beads, as previously reported in [28]).

### 2.7. Scanning Electron Microscopy

Scanning electron microscopic studies of RBC were performed by means of a JEOL JSM 5200 electron microscope. Blood samples were fixed in phosphate-buffered (pH 7.2–7.4) 2.5% glutaraldehyde for 1 h, washed two times in 0.1 M phosphate buffer (pH 7.2–7.4), and mounted on poly-L-lysine-coated glass slides. The glass slides were kept in a moist atmosphere for 1 h, washed in phosphate buffer, postfixed in 1% osmium tetroxide for 1 h, rinsed in distillated water, and dehydrated in graded ethanol (50%–70%–90%–100%). After critical-point drying with liquid CO_2_ in a vacuum apparatus and covering with a gold-palladium layer, the samples underwent scanning electron microscopic analysis and classification between reversible and irreversible phenotypes, as in the work of D'Alessandro et al. [9]. The percentages of discocytes, echinocytes, spheroechinocytes, stomatocytes, spherostomatocytes, and spherocytes were evaluated by counting 1000 to 1500 cells in randomly chosen fields. In detail, as reported in [9], RBCs manifesting echinocyte and stomatocyte shapes are capable of returning to the discocyte shape under certain conditions. Thus, these RBC shape changes are considered potentially reversible transformations. In contrast, RBCs assuming spheroechinocyte, spherostomatocyte, spherocyte, ovalocyte, and degenerated shapes are irreversibly changed cells.

## 3. Results and Discussions

Currently, whole blood withdrawn from a single donor (*≈*450 mL) is collected in a CPD anticoagulant-containing plastic (usually bis(2-ethylhexyl)phthalate-DEHP) multiple blood-pack units. In each system, the main unit (where blood is at first collected during withdrawal) contains 63 mL of CPD solution. Three satellite units (for platelet concentrates, plasma, and erythrocyte concentrates) are also present, where cellular components are split upon centrifugation at 1500 rpm for 10 min. The satellite unit for erythrocyte concentrates contains 100 mL of SAGM as additive solution. Most commonly available centrifuges can load up to six plastic bags for each run. Plastic bags are, then, put in a phase separator and RBCs present in the bottom of the bag are sterilely transferred through a plastic tube in the final SAGM-containing plastic bag and shipped for hypothermic storage. Before the cycle might start again with six new units, plasma and platelets require additional centrifugation steps, leaving a time frame for additional limited manipulation on EC alone. Any ideal further step to be introduced in the blood components production chain with the goal to reduce storage-induced lesions [8–12], eventually including also blood deoxygenation [13, 19–21], should be designed as to fit these preparation cycles. In our opinion, this could be best obtained by performing deoxygenation of erythrocyte concentrates in the time window ranging from the separation of blood components through centrifugation of six units to the next cycle of six units.

The aim of this technical report is to demonstrate the feasibility of a rapid and efficient deoxygenation method to be eventually introduced in clinical routine practice, whether large-scale laboratory and clinical trials will outline any significant improvement of deoxygenated RBC concentrate units over current “aerobically” stored counterparts. The deoxygenation workflow would take place in 30 minutes and does not require any transfer of erythrocytes to additional satellite units nor does it compromise safety and effectiveness of the blood therapeutic [9, 19–21]. The components of the deoxygenation apparatus (Figure 1) are relatively inexpensive (less than 2,000 €, subject to regional sales oscillations). 

Helium was chosen to perform deoxygenation, since it is an inert gas, which can be easily found at the highest commercial purity. Attempts to perform deoxygenation were carried on with other gases, including Argon (expensive) and nitrogen. The latter is more difficult to be extracted from air (or commercially obtained) at the highest purity and was excluded for two main reasons: (i) even after performing multiple cycles of deoxygenation, we could still observe a 6%–10% residual oxyhaemoglobin; (ii) after twenty-eight days of storage under nitrogen, we could observe a significant haemolysis, which we interpreted as a nitrogen radical species-(RNS-) triggered phenomenon. 

It could be argued that helium represents a limited resource, other than a rather expensive one. In this view, we care to stress that one single highest purity helium cylinder would theoretically suffice to perform deoxygenation of thousands of units. Although we do understand that this does not solve the issue related to the limitedness of the helium resource, this is a concern that would involve any other technology that currently adopts heliumas the working gas, such as collision-induced dissociation (CID) mass spectrometry. While these instruments have become increasingly widespread, helium based CID still represents one key approach to investigate, for example, protein, peptide, and metabolic species at the molecular level. Alternative strategies might suggest to rely upon other noble gases for deoxygenation of RBCs (such as Argon) which, however, would not make it any better in terms of availability of the raw material (in this case gas) in the long-term and on a larger scale.

As for the deoxygenation procedure, it could be argued that simple flushing with gases would easily replace the timed valve system. We could experience that after one-hour flushing we could not obtain full deoxygenation, which was instead rapidly achieved through multiple repeated cycles of gas influx/efflux through timed valves (upon manual optimization of optimal gas exchange rates). Besides, flushing resulted in the evaporation of liquid components (additive solution) altering the osmolarity of the solution and influencing RBC physiology and, inevitably, morphology. Conversely, the maintenance of temperature homeostasis through the heated stainless steel unit holder and gentle agitation, along with aspiration (without reaching vacuum as RBC would hemolyse), dramatically improved rapidity and efficiency of the deoxygenation protocol without altering RBC integrity and functionality.

It is worthwhile to note that although the Flick law would suggest to use larger plastic units for storage of ECs in order to increase the surface available for gas exchange and, thus, increase the rapidity of the deoxygenation process, in the hereby proposed method, we could obtain rapid deoxygenation with commercially available plastic bags. However, owing to plastic bag permeability to gases, in order to maintain deoxygenation of the units, we stored deoxygenated in units in helium chamber under controlled conditions.

One additional parameter that could be implemented in the process would be the acidification of pH in order to reduce haemoglobin oxygen affinity during deoxygenation. This would imply that pH should be buffered towards alkalinization later on, since an alkaline pH is known to improve RBC viability via the upmodulation of metabolism (ATP and 2,3 DPG are maintained for a longer period in alkaline solutions [29]). Though we also tried to perform deoxygenation at an acidic pH (through buffering via acidic citrate), we did not observe any significant improvement in the workflow except for the rapidity of the deoxygenation process, which was obviously faster. On the other hand, it should be also noted that deoxygenation itself promotes the alkalinization of the medium, through oxygen and bicarbonate ion removal. While it is beyond the scope of this technical note to propose alternative additive solutions, it could be feasible enough to perform deoxygenation on RBCs collected in acidic pH CPD (or CP2D), centrifuged, and leukofiltered, prior to the transfer into a satellite unit containing a more alkaline pH commercial solutions (such as phosphate-adenine-glucose-guanosine-gluconate-mannitol (PAGGGM) [29]). 

Besides, gas impermeable plastic bags would have eased storage under deoxygenated conditions, although we could achieve this goal through locating the units in a closed chamber conditioned with helium and stored at refrigerated temperatures (1–6°C). Current plastics, of which commercially available EC storage bags are made, hold some advantages as well, such as that they do not hamper spectrophotometric absorbance assays in the 500–600 nm range, as they can be adjusted vertically as to screen haemoglobin absorbance curve without any significant scattering or interference (Figure 2). Of note, we also tried to adopt oxygen sensors to monitor oxygen levels in the unit upon each deoxygenation cycle. These optic chemosensors detect the presence of oxygen through monitoring the quenched or reduced fluorescence of a specific fluorophore (polydimethylsiloxane) with a long excited-state lifetime upon collision with oxygen molecules [30]. The lower the oxygen pressures are, the lower is the likelihood of collisions between oxygen molecules and the fluorophore, which is reversely measured through fluorescence detection [30]. Unfortunately, these sensors (and the fluorophore-coated surfaces, which are meant to be inserted into the plastic bag where blood is stored) are rather expensive, and their safety in the frame of direct contact with transfusable RBCs is yet to be ascertained. Conversely, haemoglobin is totally costless and as much as informative. Within the framework of the present report, when spectrophotometric assays of haemoglobin indicated complete deoxygenation, further testing with dissolved oxygen sensors by tryptophan fluorescence quenching indicated pO_2_ below 1 ppb (<0.0021 mmHg) (Steroglass, Perugia, Italy), below the limit of detection of the instrument.

### 3.1. A Small-Scale Study on the Effectiveness of Deoxygenation on Long-Term Storage: Preliminary Results

While the hereby reported method is only designed as a proof of concept about the feasibility of the deoxygenation approach, we also performed a preliminary small-scale study to collect preliminary indicative (albeit not significant, since the power of the study would not allow to draw any biologically meaningful and reliable conclusion) evidences about the effects of deoxygenation on RBCs storage. 

In order to determine whether the deoxygenation treatment resulted in alterations of RBC morphology and efficiency, we first tested the rapidity of the reoxygenation process. We could obtain re-oxygenated haemoglobin after less than one minute of exposure to environmental oxygen conditions. Therefore, reoxygenation of blood before its usage does not require any additional manipulation, as it would rapidly occur naturally *in vivo* during the slow process of transfusion to the recipient.

**Figure 3:**
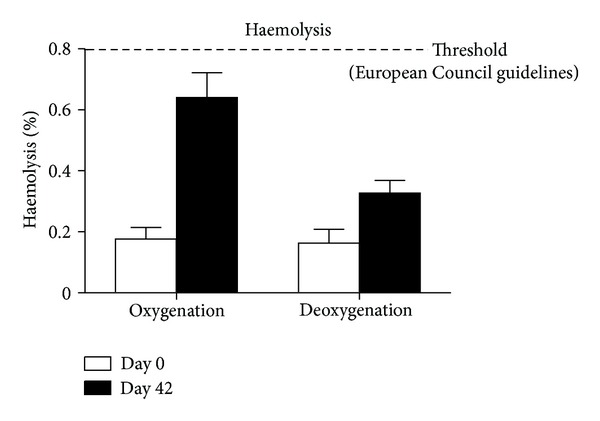
Haemolysis levels in control (left columns) red blood cells and red blood cells after deoxygenation (right columns) at day 0 (white columns) or after 42 days (black columns) of refrigerated liquid storage. At day 0, deoxygenated red blood cells do not show any significantly increased haemolysis. On the other hand, haemolysis is lower in deoxygenated red blood cells than in controls after 42 days of storage.

As for RBC integrity, no significant haemolysis was observed after deoxygenation treatments (0.17 + 0.04% and 0.16 + 0.04% in controls and deoxygenated units at day 0, resp.), while haemolysis in 42-day stored ECs under helium was significantly lower than that in controls (0.33 + 0.04% versus 0.64 + 0.08%, respectively, *P* value <0.05 ANOVA) (Figure 3).

Hereby reported preliminary results also seem to suggest that the storage of ECs under helium results in a reduced vesiculation likelihood (in Table 1, we report the counted events within the defined 5-minute time window in the gated area for side scatter and forward scatter for day 42 controls and deoxygenated counterparts). Although further more in depth investigations are mandatory, the observation about an apparent decreased vesiculation rate of deoxygenated RBCs is in agreement with previous reports by Yoshida's group [19–22].

**Figure 4:**
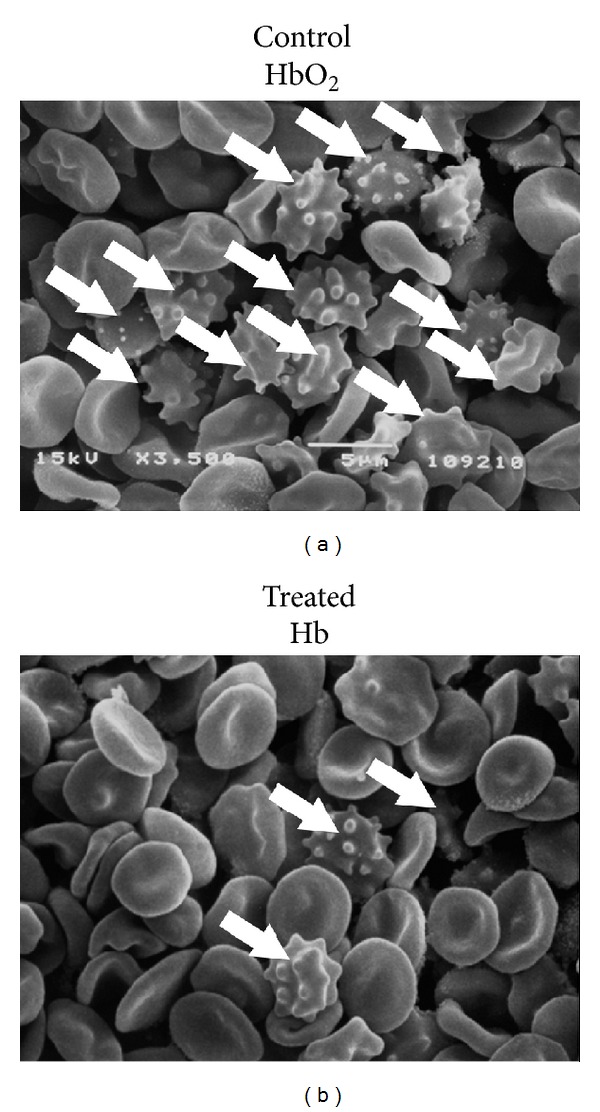
The extent of membrane shape alterations is lower in deoxygenated (b) than in control (a) red blood cells after 42 days of storage, as it emerged from preliminary scanning electron microscope (SEM) analysis.

Deoxygenation also apparently resulted in a narrower extent of the morphology alteration phenomena. We could indeed observe that while 42-day old controls displayed almost 80% of either reversibly (echinocytes and stomatocytes) and irreversibly (spheroechinocyte, spherostomatocyte, spherocyte, ovalocyte, and degenerated shapes) altered RBCs and only 20.6 ± 2.5 discocytes, the deoxygenated long stored counterparts conserved a greater percentage of unaltered discocytic phenotypes (*≈*32.1%) and of reversibly modified RBCs and a lower percentage of irreversibly altered erythrocyte shapes (Table 2). It is worthwhile to stress that whether larger-scale studies will confirm these results, it would be possible to conclude that shape-based classification of deoxygenated RBCs closely resembles control RBCs stored for a shorter period (28–35 days), in relation to the values that we could previously report for untreated CPD-SAGM control erythrocytes [9, 31]. We also provide a snapshot of this phenomenon in Figure 4, which shows a scanning electron microscope-(SEM-) obtained micrograph of RBCs from EC units stored in presence (a) or in absence of oxygen (b); arrows indicate RBCs showing irreversibly altered morphologies, as previously reported [9].

An in-depth laboratory investigation is currently in progress which aims to assess the potential benefits of deoxygenated storage, including the monitoring of several parameters such as RBC morphology, vesiculation, and alterations to the membrane proteome (*paper in preparation*). On the other hand, we recently proposed a detailed study about the alterations to the metabolic fluxes upon deoxygenation and over storage duration on a weekly basis [32] and compared the results to our in-depth analyses on CPD-SAGM-stored untreated RBCs [33]. From this study, it emerged that the deoxygenation of RBCs might result in the alteration of the redox poise by upmodulating the nitric oxide (NO) metabolism, which is known to influence the production of RNS, and by blocking the oxidative stress-triggered metabolic diversion from the Emden Meyerhof classic glycolytic pathway towards the pentose phosphate pathway, which should instead provide reduced coenzymes to regenerate the antioxidant battery, such as NADPH) [31].

## 4. Conclusion

While the clinical improved efficiency of deoxygenated ECs it is yet to be demonstrated, preliminary laboratory evidences [12, 19–21] seem to suggest that deoxygenation might soon become a critical step in the transfusion service pipelines. To this end, we hereby proposed a cheap apparatus for rapid and effective blood deoxygenation for transfusion purposes. We demonstrated its straightforward setup and functioning principles. Also, through a small-scale preliminary study, we report the effectiveness of this method in delivering deoxygenated RBCs, which do not show any substantial hemolysis after handling while they show improved morphology homeostasis maintenance and reduced vesiculation after 42 days of storage. However, since the hereby presented is but a methodology paper, it is worthwhile to stress that further more in-depth and larger-scale investigations are encouraged in order to draw any biologically meaningful conclusion. 

## Figures and Tables

**Table 1:** RBC-shed microparticles.

Storage day	Microparticles (counted events in the arbitrary time window inside the gated area)
42 (control)	5234 ± 125
42 (deoxygenated)	1865 ± 78

**Table 2:** SEM erythrocyte shape classification.

Storage day	Discocyte (%)	Reversibly* changed RBC (%) (echinocyte and stomatocyte shapes)	Irreversibly* changed RBC (%) (spheroechinocyte, spherostomatocyte, spherocyte, ovalocyte, and degenerated shapes)
0	76.5 + 3.1	19.2 ± 5.7	4.3 ± 2.6
42 control	20.6 ± 2.5	43.2 ± 3.8	36.2 ± 2.9
42 deoxygenated	32.1 ± 1.9	45.4 ± 2.2	22.5 ± 3.1

*Reversible and irreversible changes were classified based on classification criteria, as previously reported by D'Alessandro et al. [9].
